# Porcine antiviral activity is increased by CRISPRa-SAM system

**DOI:** 10.1042/BSR20191496

**Published:** 2019-08-19

**Authors:** Jinhe Jiang, Yumei Sun, Rong Xiao, Kai Wai, Muhammad Jamil Ahmad, Faheem Ahmed Khan, Hongbo Zhou, Zhiyong Li, Yong Zhang, Ao Zhou, Shujun Zhang

**Affiliations:** 1 Key Laboratory of Agricultural Animal Genetics, Breeding and Reproduction Ministry of Education, Huazhong Agricultural University, Wuhan 430070, Hubei, People’s Republic of China; 2 State Key Laboratory of Agricultural Microbiology, Huazhong Agricultural University, Wuhan 430070, Hubei, People’s Republic of China; 3 The Center for Biomedical Research, Key Laboratory of Organ Transplantation, Ministry of Education, Ministry of Health, Tongji Hospital, Tongji Medical College, Huazhong University of Science and Technology, Wuhan 430070, Hubei, People’s Republic of China; 4 State Key Laboratory of Veterinary Etiological Biology, Lanzhou Veterinary Research Institute, Chinese Academy of Agricultural Sciences, Lanzhou, Gansu, People’s Republic of China; 5 Department of Veterinary Medicine, Gansu Agricultural University, Lanzhou, People’s Republic of China; 6 Department of Medical Cell Biology and Genetics, Southwest Medical University, Luzhou, Sichuan, People’s Republic of China; 7 Wellcome Genome Campus, Wellcome Sanger Institute, Hinxton, Cambridgeshire, United Kingdom

**Keywords:** B4galnt2, CRISPRa-SAM system, Mx2, Porcine antiviral activity

## Abstract

Clustered Regularly Interspaced Short Palindromic Repeat activation-synergistic activation mediator system (CRISPRa-SAM) has been efficiently used to up-regulate the targeted genes in human and mouse. But it is not known whether the CRISPRa-SAM system can be used against porcine disease because its two important transcriptional activation domains (P65 and heat shock transcription factor 1 (HSF1)) are from mouse and human, respectively. Pig is one of the most important meat sources, porcine viral infectious diseases cause massive economic losses to the swine industry and threaten the public health. We aimed to investigate whether the CRISPRa-SAM system could increase porcine antiviral activity by mediating two pig-specific target genes (*Mx2* and β1,4 N-acetylgalactosaminyltransferase (*B4galnt2*)). First, we constructed PK-15 and IPEC-J2 cell lines expressing nuclease-deficient Cas9 (dCas9)-vp64 and MS2-P65-HSF1 stably. Next, in these two cell models, we activated *Mx2* and *B4galnt2* expression through CRISPRa-SAM system. Antiviral activity to PRV or H9N2 was improved in PK-15 cells where *Mx2* or *B4galnt2* was activated. Altogether, our results demonstrated the potential of CRISPRa-SAM system as a powerful tool for activating pig genes and improving porcine antiviral activity.

## Introduction

Pig is one of the most important meat producing animals. Porcine viral infectious diseases cause massive economic losses to the swine industry. Enhancing the antiviral ability of pigs has been a keen interest for researchers. Zhao et al. (2107) reported to reduce the fat deposition, and increase lean meat production in porcine, through reconstitution of *UCP1* using CRISPR/Cas9 [1]. Burkard et al. (2017) used CRISPR/Cas9 to produce pigs completely resistant to porcine reproductive and respiratory syndrome virus (PRRSV) by removing the CD163 subdomain 5 [2].

Nonetheless the Clustered Regularly Interspaced Short Palindromic Repeat activation-synergistic activation mediator system (CRISPRa-SAM) system needs to be explored for pig antiviral breeding. This system is an extension of the conventional CRISPR knockout system. Instead of wild-type Cas9, designed variants of a nuclease-deficient Cas9 (dCas9) carrying the D10A and H840A mutations are used in the CRISPRa-SAM system. The dCas9 is fused to herpes simplex virus (HSV) viral protein 16 (VP16) activation domains (known as VP64) and the system includes some synergistic activation mediators. These mediators comprise fusion proteins (MS2-guide RNA (gRNA), dCas9-VP64 and the MS2-P65-HSF1), MS2 bacteriophage coat protein fused to the NF-κB (P65) and heat shock transcription factor 1 (HSF1) [3–5]. It is widely used to up-regulate genes in human cells [6–8], rats [9,10] and fruit flies [11]. The CRISPRa-SAM system’s activation domains P65 and HSF1 are from mouse and human, so it is required to reveal the role of CRISPRa-SAM as a gene regulator in pig cells for porcine antiviral activity.

Myxovirus resistance proteins (Mx) are a family of dynamin-like GFPases, and can inhibit influenza A virus. In mammals, *Mx* gene has two isoforms, *Mx1* and *Mx2. Mx1* can cause hindrance in replication of RNA and DNA viruses [12]. *Mx2* has a tendency to affect the anti-human immunodeficiency virus type 1 (HIV-1) activity of type I IFN [13,14]. Meanwhile, *Mx2* can inhibit PRRSV infection in pig [15] and herpes virus infection [16]. Glycosylation is one of the most important post-translational modifications of proteins in eukaryotic cells. In the Golgi, terminal glycosylation reactions generate a huge panel of glycan that confer a variety of structural and functional roles to the glycoproteins exposed at the cell surface. β1,4 N-acetylgalactosaminyltransferase (B4GALNT2) catalyzes the last step in the biosynthesis of the human Sd(a) antigen through the addition of an N-acetylgalactosamine residue via a β-1,4 linkage to a subterminal galactose residue substituted with an α-2,3-linked sialic acid [17,18]. Recently, a study suggested that B4GALNT2 overexpression can prevent the infection of every avian influenza virus strain [19]. So we chose these two pig genes for the present study.

In the present study, we evaluated the ability of the CRISPRa-SAM activation systems to activate *Mx2* and *B4galnt2* gene expression in two kinds of porcine cell lines (PK-15, IPEC-J2). We observed PK-15 cells had more antiviral activity to PRV or H9N2 when *Mx2* or *B4galnt2* expression was activated. The present study highlights that biotechnology has great potential to manipulate pig breeding for improving porcine antiviral activity.

## Materials and methods

### Plasmids construction

The location of the transcription start site (TSS) of sgRNA is very important to cause activation of genes. The design of the sgRNA was based on the principle around the upstream of the target gene TSS followed the GN (19) NGG. Four sgRNAs of each gene were designed and synthesized, named as sgRNA1, sgRNA2, sgRNA3 and sgRNA4, respectively (Figure 2). The sgRNAs used in the present study were designed by http://crispr.mit.edu/, and are listed in Table 1.

**Table 1:** gRNA sequences of two pig genes (*Mx2, B4galnt2*)

Target gene	Number	Guide sequence	TSS distance
***Mx2***	1	CTGGAAGGGAGGTACACCA	−61
	2	TCGGGAAGAGGGCACATTC	+170
	3	GACGAGCCATAGATGCGTGC	−1
	4	ATTCTTGAGTTTCATTTCT	−112
***B4galnt2***	1	CGTGGCGGTTCGGTTCGTGG	−87
	2	CAGTCCCGGCTTACGGCAC	−202
	3	GGAGAGGCCGAACCGCCAC	−137
	4	GCGTCCGAGTTGATGCAAG	−428

Oligomers were synthesized for all sgRNA sequences, annealed and cloned into lenti-sgRNA (MS2)-pure backbone (#73795) using *Bbs*I digestion. Sequencing for all plasmids was done before use.

### Cell culture

PK-15 cells (pig kidney cell line), IPEC-J2 cells (pig intestinal epithelial cell line) and HEK293T cells were maintained in DMEM supplemented with 10% fetal bovine serum (FBS) and were cultured in an incubator at 37°C with 5% CO_2_.

### Generation of PK-15/J2-CRISPRa-SAM stable cell line and transfection

HEK293T cells were used for generating lentiviruses expressing dCas9-VP64 and MS2-P65-HSF1 by co-transfection of the packaging plasmids psPAX.2, pMD2.G and lentidcas9-VP64_blast (#61425) or lentiMPHv2 (#89308) using Neofect transfection reagent (Neofect Biotech, China). Supernatant containing virus was harvested at 48 and 72 h post transfection. PK-15 or IPEC-J2 cells were exposed to virus with polybrene (8 μg/ml; Sigma–Aldrich) and selection was done using blasticidin (6 μg/ml; InvivoGen, Thermo Fisher) and hygromycin (300 μg/ml; Sangon Biotech). Expression of SAM components was tested using RT-qPCR. The cell lines successfully expressing the SAM components were named ‘PK15-CRISPRa-SAM and J2-CRISPRa-SAM’, respectively.

PK15-CRISPRa-SAM cells or J2-CRISPRa-SAM cells were seeded into 12-well plates for transfection. The cells were exposed to lentiviruses loaded *Mx2* or *B4galnt2* sgRNAs with polybrene (8 μg/ml; Sigma–Aldrich). After 48 h of infection, the cells were selected using puromycin (3 μg/ml; Gibco, Thermo Fisher) for at least 14 days while replacing the puromycin every 3 days.

### Quantitative RT-PCR analysis

Total RNA kit I (Omega, U.S.A.) was used for total RNA collection following the manufacturer’s instructions. Total RNA in equal volumes were reverse transcribed for cDNA using RevertAid First-Strand cDNA Synthesis Kit (Thermo Fisher Scientific, Baltic, U.S.A.). Real-time PCR was performed (CFX96 Real-Time System; Bio-Rad) for all the samples in duplicate and data were normalized to β-actin. Thermal cycling conditions were 10 min at 95°C and 40 cycles of 10 s at 95°C, 30 s at 60°C and 10 s at 72°C. *Mx2* forward primer is 5-CCGAGAAAGTTGTCCTGAATGTG-3, reverse primer is 5-TGCGGATGCGAGTGAAAGAAT-3. *B4galnt2* forward primer is 5-GCGACTCCAAAGAATTGGCTTC-3, reverse primer is 5-TGGTGACCTATGATCACGTGTG-3. β-actin forward primer is 5- TGGCACCACACCTTCTACA-3, reverse primer is 5-ATCTTCTCACGGTTGGCTTTG-3.

### Virus titration

PK15-CRISPRa-SAM cells and PK-15 cells with activated (ten-fold) *B4galnt2* gene were infected with A/chicken/Shanghai/SC197/2013 (SH13, H9N2) at an MOI (0.001 or 0.01). A/chicken/Shanghai/SC197/2013 (SH13, H9N2) was gifted by Professor Chenjun Li from Harbin Veterinary Research Institute, Chinese Academy of Agricultural Sciences. Viral supernatants were harvested post-infection at the indicated time points for hemagglutination assay to titrate virus titer as described previously [20].

### Virus infection

PK15-CRISPRa-SAM cells and PK-15 cells with activated (600-fold) *Mx2* gene were infected with PRV BAC (pBecker2) at an MOI (100 or 10) for 24 h. pBecker2 was gifted by Professor Hanzhong Wang from Wuhan institute of Virology, Chinese Academy of Sciences. Fluorescence microscopy (Carl Zeiss, LSM 800) was used to observe under a 20× objective.

### Statistical analysis

All the experiments were run three times, and results were documented as means ± SEM. The qPCR results were analyzed by two-way ANOVA in GraphPad Prism 6.01, while other results were analyzed by Student’s *t* test in GraphPad Prism 6.01. Statistical significance was defined by a *P*-value of less than 0.05.

## Results

### The transcriptional activation domains of pig P65 and HSF1 genes were highly homologous with that of P65, HSF1 of the CRISPRa-SAM system

**Figure 1:**
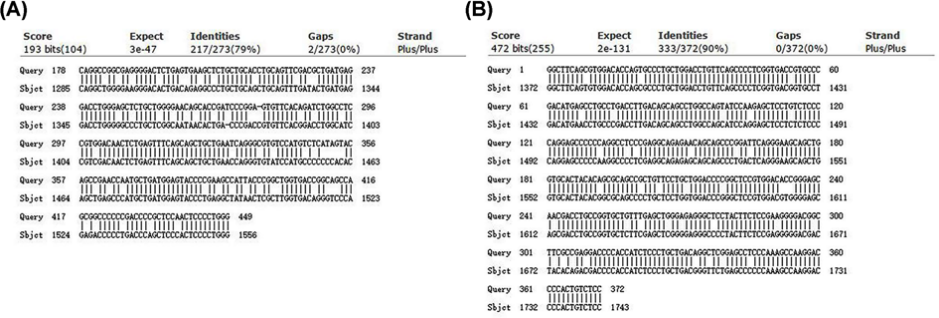
Comparison of transcriptional activation domains sequences in CRISPRa-SAM system and pig gene sequences (**A**) Comparison of p65 sequences in CRISPRa-SAM system and pig p65 gene. (**B**) Comparison of HSF1 sequences in CRISPRa-SAM system and pig HSF1 gene. The lower sequences were from pig HSF1 and p65, and the upper sequences were from human HSF1 and murine p65 as transcriptional activation domains in CRISPRa-SAM system.

First, we checked whether the transcriptional activation domains of pig (P65, HSF1) are homologous to the activation domains (P65, HSF1) of the CRISPRa-SAM system. Results computed by NCBI blast showed pig P65 (FN999988.1) and HSF1 (XM_005655310.3) were highly homologous to that of CRISPRa-SAM system. The identity and gap for HSF1 in pig were 89.37% (311/348) and 6.45% (24/372), respectively (Figure 1), while those of P65 were 71.03% (385/542) and 2.34% (13/555), respectively (Figure 1). It is worth mentioning that the efficacy of CRISPRa-SAM system has not been validated in pigs before.

**Figure 2:**
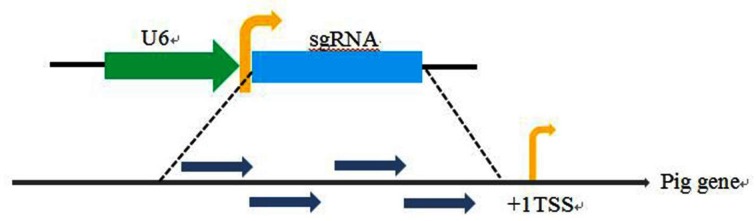
The strategy of design the sgRNA of target pig gene Single-gRNA (sgRNA) scaffold was under the control of a U6 promoter. The sgRNAs (sgRNA1, sgRNA2, sgRNA3 and sgRNA4) were located to the upstream of TSS of the target gene.

### The important components of CRISPRa-SAM system efficiently expressed in two pig cell lines

We checked the expression till day 14 post selection, for important components of CRISPRa-SAM system by infecting PK-15 and IPEC-J2 cells with lentiviruses expressing dCas9-VP64 and MS2-P65-HSF1, respectively. The result showed that dCas9-VP64 and MS2-P65-HSF1 were stably and effectively expressed in two pig cell lines. *C*_t_ values of dCas9-VP64 and MS2-P65-HSF1 were 22.14 + 0.79 and 21.65 + 0.11, respectively in the PK15-CRISPRa-SAM cell line, meanwhile *C*_t_ values of dCas9-VP64 and MS2-P65-HSF were 21.44 + 0.01 and 21.24 + 0.05 in the J2-CRISPRa-SAM cell line, respectively (Tables 2 and 3).

**Table 2:** Expression of SAM components in the PK15-CRISPRa-SAM cell line

Gene	PK-15 *C*_t_ values	PK15-CRSIPRa-SAM *C*_t_ values	*P*-value
***dCas9-vp64***	28.94 + 0.63	22.14 + 0.79	>0.0001
***Ms2-p65-HSF1***	+35	21.65 + 0.11	>0.0001
***β-actin***	18.67 + 0.18	19.37 + 0.11	0.04

**Table 3:** Expression of SAM components in the J2-CRISPRa-SAM cell line

Gene	IPEC-J2 *C*_t_ values	J2-CRSIPRa-SAM *C*_t_ values	*P*-value
***dCas9-vp64***	28.55 + 0.49	21.44 + 0.01	>0.0001
***Ms2-p65-HSF1***	+35	21.24 + 0.05	>0.0001
***β-actin***	18.31 + 0.19	18.12 + 0.0	0.14

### CRISPRa-SAM system could effectively activate transcription in different pig cell lines with different efficiencies

**Figure 3:**
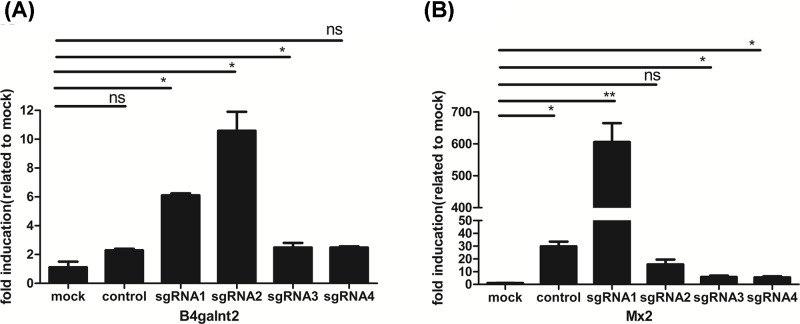
Activation of target pig gene in pig PK15 cells by CRISPRa-SAM system (**A**) Related mRNA expression of pig *B4galnt2*. (**B**) Related mRNA expression of pig *Mx2*. Lentivirus expressing gRNA of target pig gene infected PK15-CRISPRa-SAM cells, the cells were selected with 3 μg/ml puromycin for 14 days. Related mRNA expression of target genes were detected by RT-qPCR**.** Lentivirus expressing gRNA of no target gene was used to as the control, PK15-CRISPRa-SAM cells were mock. The data were represented as the mean ± SEM (*n*=3). Statistically significant differences were determined by two-way ANOVA, ns > 0.05, *P<0.05, **P<0.01.

**Figure 4:**
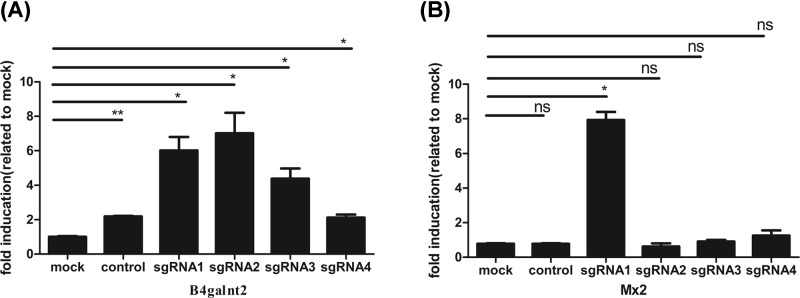
Activation of target pig gene in pig IPEC-J2 cells by CRISPRa-SAM system (**A**) Related mRNA expression of pig *B4galnt2*. (**B**) Related mRNA expression of pig *Mx2*. Lentivirus expressing gRNA of target pig gene infected IPEC-J2-CRISPRa-SAM cells, the cells were selected with 3 μg/ml puromycin for 14 days. Related mRNA expression of target genes were detected by RT-qPCR**.** Lentivirus expressing gRNA of no target gene was used as the control, IPEC-J2-CRISPRa-SAM cells were mock. The data were represented as the mean ± SEM (*n*=3). Statistically significant differences were determined by two-way ANOVA, ns > 0.05, *P<0.05, **P<0.01.

We designed the gRNAs of *Mx2* and *B4galnt2* through the website http://crispr.mit.edu/. These gRNAs are listed in Table 1. After the lentivirus expressing gRNAs of *Mx2* or *B4galnt2* infected PK15-CRISPRa-SAM cell line or J2-CRISPRa-SAM cell line, we checked the activation of pig genes (*Mx2* and *B4galnt2*) expression using CRISPRa-SAM system in two different pig cell lines (PK-15 and IPEC-J2). The results showed up-regulation of *Mx2* and *B4galnt2* genes in both (PK-15 and IPEC-J2) pig cell lines (Figures 3 and 4). Up-regulation of activated *Mx2* was 600- and 10-fold in pig cells PK-15 and IPEC-J2, respectively, while activated *B4galnt2* gene was up-regulated by 10-fold in PK-15 cell line and 7-fold in IPEC-J2 cell line (Figures 3 and 4).

### Antiviral activity was increased in PK-15 cells

**Figure 5:**
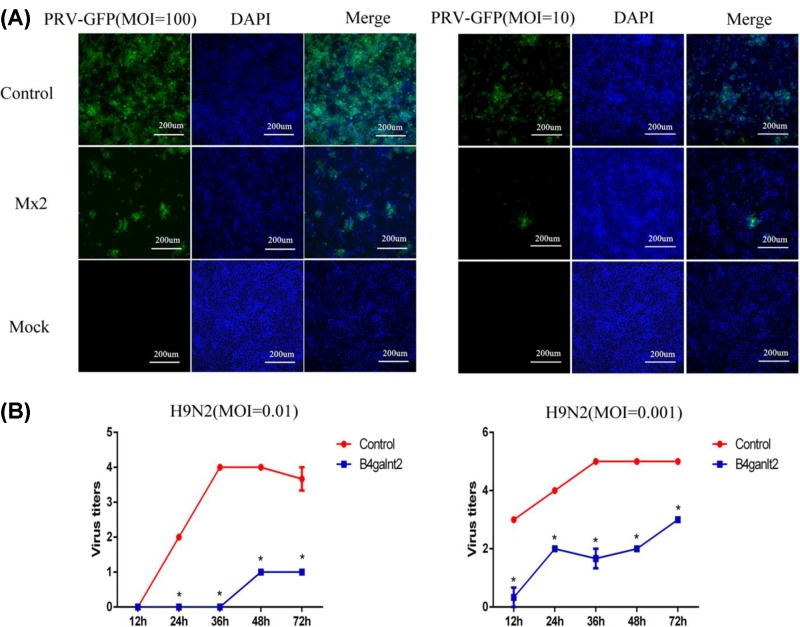
Activated *Mx2* and *B4galnt2* improved their antivirus functions (**A**) PK15-CRISPRa-SAM cells (control) or PK-15 cells in which *Mx2* gene was activated with 600-fold were infected with PRV-GFP at an MOI of 100 or 10, 24 h after infection, fluorescence microscopy microscopy was used to observe them (200×). (**B**) PK15-CRISPRa-SAM cells (Control) or PK-15 cells in which *B4galnt2* gene was activated by ten-fold were infected with H9N2 virus at an MOI of 0.01 and 0.001. Viral supernatants were harvested at the indicated time points post infection and hemagglutination assay to titrate virus titer. Statistically significant differences were determined by Student’s *t* test, *P<0.05.

*Mx2* gene has broad-spectrum antiviral effect, hence the overexpression of *Mx2* could inhibit DNA and RNA viruses. To check this hypothesis, up-regulation of pig genes mediated by the CRISPRa-SAM system in pig cells could improve their normal functions, we tested the *Mx2* expression level with relation to its antiviral activity against Pseudorabies virus (PRV) (MOI = 100 or 10) by infecting PK-15 cells activated *Mx2* approximately 600-fold higher by CRISPRa-SAM system and PK-15 cells. Results shown in Figure 5A illustrate that PRV replication was inhibited in PK15 cells after overactivation of *Mx2.*

For further investigations, antiviral activity *of B4galnt2* against pan-avian influenza virus infection [19] was checked for any increase by infecting the PK-15 cells activated *B4galnt2* approximately ten-fold higher by the CRISPRa-SAM system and PK-15 cells with H9N2 virus. The H9N2 virus is widely circulating in the world, causing rasorial infections and serving as a gene donor for H5N1, H7N9 and H10N8 viruses [21]*.* The results showed that the virus titer of the H9N2 virus produced by PK-15 cells activated *B4galnt2* was lower than the control (without gene activation) (Figure 5B). It is concluded that CRISPRa-SAM-mediated gene activation efficiently enhanced host antiviral response by increasing the expression of antiviral genes.

These results suggest that the CRISPRa-SAM system is suitable for pig gene induction and can be used in pig genome study. Nonetheless, the HSF1 and P65 used as transcriptional activation domains in CRISPR-SAM system are based on human and murine genome sources, respectively.

## Discussion

There are many advantages of the CRISPRa-SAM system over traditional overexpression gene technology. Exogenous expression vectors are used to clone cDNA sequences for traditional gene overexpression that is hard to achieve when the target gene has a long sequence or is rich in GC contents.

The CRISPRa-SAM system is capable of activating the target gene with only an sgRNA regardless the size of target gene. The CRISPRa system has been shown to activate target genes in many species [6,9,22]. Gain-of-function screening using a pool of sgRNA libraries, has been a powerful and effective tool for target gene screening [3,5,19,23–25] while cDNA library overexpression still retains some problems [3,5,19,23–25].

In the present study, we investigated the ability of the CRISPRa-SAM system to activate endogenous pig gene expression for porcine antiviral activity. Our results suggested that the CRISPRa-SAM system can effectively activate the pig endogenous gene transcriptional expression in both pig PK-15 and IPEC-J2 cell lines with increased porcine antiviral activity. We concluded that the CRISPRa-SAM system could effectively activate transcription in different pig cell lines with different efficiencies. The effect of CRISPRa-SAM was higher in PK-15 cells with 600 fold up-regulation of sgRNA1 for *Mx2*, compared with ten-fold in the IPEC-J2 cell line (Figures 3 and 4), while *B4galnt2* gene up-regulation was ten-fold in PK-15 cells vs seven-fold in IPEC-J2 cells. In a pool of four sgRNAs, the effect of sgRNA1 of *Mx2* gene had significantly higher efficiency than three others in PK-15 cells and IPEC-J2 cells and the effect of sgRNA2 and sgRNA1 of *B4galnt2* were significantly higher than others in both the cells. The porcine antiviral activity to PRV or H9N2 was improved in PK-15 cells where *Mx2* or *B4galnt2* was activated.

Similarly, in human cell lines, the efficiency of CRISPRa system to activate different target genes varied with different sgRNA. Konermann et al. (2015) [3] reported fold activation of 12 different genes (*VEGFA, HBG1, TERT, IL-1B, IL-1R2, ZFP42, MYC, LIN28A, SOX2, NANOG, KLF4, POU5F1*) mediated by the CRISPRa-SAM system and plotted against the sgRNA location in 293FT cells. In another study, a panel of validated sgRNAs was used to target the promoters of both coding (*TTN, RHOXF2, ASCL1, HBG1*) and non-coding (*MIAT, TUNA*) genes in HEK293 cell, MOLM14 cell, K562 cell and HIL60 cell, and found sgRNAs gave the highest activation in MOLM14 cell [24]. Conclusively, the efficiency of CRISPRa-SAM system is highly dependent on the cell type, target gene basal expression level and the location of gRNA. Porcine *Mx2* and *B4galnt2* were activated by CRISPRa-SAM system and porcine antiviral capacity was increased because target genes were activated in pig cells. CRISPRa-SAM system is a powerful tool to activate the expression of pig endogenous genes. The development of these pig cell models and results are not only valuable for the genome-wide screening of antiviral pig genes but also are the foundation for pig antiviral breeding with the CRISPRa-SAM system.

## Abbreviations

B4GALNT2: β1,4 N-acetylgalactosaminyltransferase
CRISPRa-SAM: Clustered Regularly Interspaced Short Palindromic Repeat activation-synergistic activation mediator system
dCas9: nuclease-deficient Cas9
gRNA: guide RNA
HSF1: heat shock transcription factor 1
IFN: interferon
MOI: multiplicity of infection
PRRSV: porcine reproductive and respiratory syndrome virus
TSS: transcription start site
